# Synchronous tuberculosis, Epstein-Barr virus-associated lymphoproliferative disorder and cytomegalovirus infection in an allogeneic transplant recipient: a case report

**DOI:** 10.1186/2193-1801-3-278

**Published:** 2014-06-02

**Authors:** Benjamin Nils Ostendorf, Christian Friedrich Jehn, Lam Giang Vuong, Hendrik Nogai, Philipp Guido Hemmati, Bernhard Gebauer, Olaf Penack, Igor Wolfgang Blau, Ioannis Anagnostopoulos, Renate Arnold

**Affiliations:** Department of Hematology, Oncology and Tumor Immunology, Charité University Medicine, Augustenburger Platz 1, 13353 Berlin, Germany; Department of Radiology, Charité University Medicine, Berlin, Germany; Institute of Pathology, Charité University Medicine, Berlin, Germany

**Keywords:** Allogeneic stem cell transplantation, Cytomegalovirus, Epstein-Barr virus, Post-transplant lymphoproliferative disorder, Tuberculosis

## Abstract

**Background:**

Allogeneic stem cell transplant recipients are prone to infections by various organisms. Tuberculosis (TB) represents a rare infectious complication, especially in countries non-endemic for TB.

**Case report:**

Here, we report the case of a German patient with exposure to TB decades before he was diagnosed with disseminated TB as well as synchronous Epstein-Barr virus associated lymphoproliferative disorder and cytomegalovirus infection after allogeneic stem cell transplantation for refractory acute myeloid leukemia. Tuberculostatic and virostatic therapy was administered and the patient could be discharged with no apparent signs of infection two weeks after initiation of therapy.

**Conclusion:**

This case illustrates the need for awareness of mycobacterial infections in patients from non-endemic regions undergoing stem cell transplantation even if other reasons for fever are present.

## Background

Infections are a major reason for morbidity and mortality in patients undergoing allogeneic hematopoietic stem cell transplantation (ASCT). Tuberculosis (TB) remains a major health threat in many parts of the world. However, TB is rare in non TB-endemic countries, including immunocompromised patients after ASCT with reported rates ranging between less than 1% in the US and up to 16% in Pakistan (Russo et al. 2010). For Europe, mycobacterial infections in ASCT recipients have been reported at a rate of 0.79% (Cordonnier et al. 2004). Usually, TB affecting transplant patients in countries with low TB prevalence is confined to foreign-born patients (Garces Ambrossi et al. 2005).

Diagnosis of TB has traditionally relied on microscopic detection of acid-fast bacilli and bacterial cultures. Nowadays, genomic amplification of mycobacterial nucleic acids has improved sensitivity. The diagnosis is difficult because TB infection can present with clinical and radiological signs resembling infections caused by other, more frequent pathogens, such as fungi. In addition, TB is not often considered in the initial differential diagnosis in febrile patients after ASCT due to its low incidence in this population. Here, we report the case of a German man who underwent ASCT and developed pulmonary and nodular TB in addition to pulmonary cytomegalovirus (CMV) infection and Epstein Barr-virus (EBV)-associated lymphoproliferation.

## Case description

In July 2012 a 51-year-old German male patient was diagnosed with acute myeloid leukemia with maturation. The disease proved refractory after administration of two cycles of induction chemotherapy and ASCT from a non-related donor with human leukocyte antigen-A mismatch (9/10 antigens matched) was performed as salvage therapy in December 2012. Conditioning consisted of the FLAMSA-RIC regimen (fludarabine 120 mg/m^2^, cytarabine 8,000 mg/m^2^, amsacrine 400 mg/m^2^, total body irradiation 4 Gy, cyclophosphamide 120 mg/kg and anti-thymocyte globulin (ATG, Fresenius, 60 mg/kg). Graft-versus-host disease (GVHD) prophylaxis was cyclosporine A from day -2 (target serum trough level of 180–220 μg/l) and mycophenolate mofetil from day 0 (2 g/day). Ciprofloxacin, acyclovir, voriconazole and monthly pentamidine inhalations were administered as anti-infective prophylaxis. Pre-transplant X-ray of the lungs did not show any pathologic findings and polymerase chain reactions (PCR) for CMV and EBV genomes in the peripheral blood were negative. The patient reported exposure to TB in an affected classmate decades ago but denied previous infection. BCG vaccination status was unknown. Tuberculin skin test is not routinely performed at our institution. The patient tolerated the conditioning regime well and received a non T-cell-depleted peripheral blood stem cell allograft containing 4.9 × 10^6^ CD34+ cells/kg and 76.8 × 10^6^ CD3+ cells/kg. From day +5 granulocyte-stimulating factor (5 μg/kg/day) was given intravenously until the absolute leukocyte count exceeded 1 × 10^9^/l.

On day +1 after transplantation the patient developed fever of up to 38.5°C and the antibiotic treatment was switched to meropenem. After detection of Staphylococcus haemolyticus in a blood culture, vancomycin was added and the fever subsequently subsided. On day +8 fever recurred and vancomycin was exchanged for linezolid. Computer tomography (CT) of the lungs revealed micronodular lesions suspicious of calcified granulomas but did not show signs of acute pulmonary infection. Subsequently, the fever ceased and engraftment of neutrophils was achieved on day +19.

**Figure 1:**
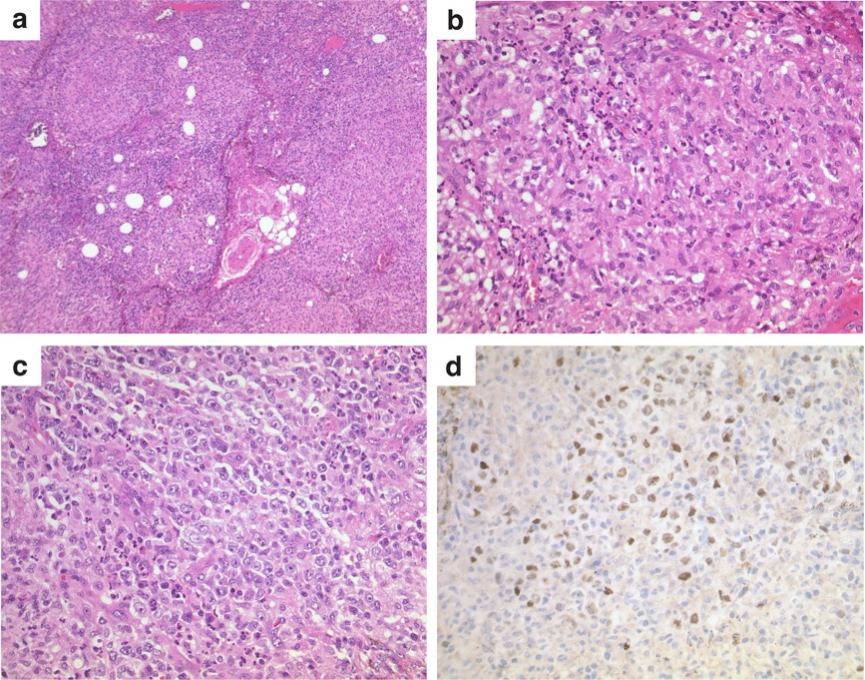
**Histological diagnosis of synchronous nodular tuberculosis and EBV-associated lymphoproliferative disorder.** Biopsy of a cervical lymph node performed on day +36 showed effacement of the architecture due to numerous granulomas (low magnification **(a)**, which were composed of epithelioid cells **(b)**. Between the granulomas a polymorphous lymphoid infiltrate was noted containing blasts as well as plasma cells **(c)**. The blasts were latently EBV-infected as shown in the immunohistochemical demonstration of EBNA2 **(d)**.

**Figure 2:**
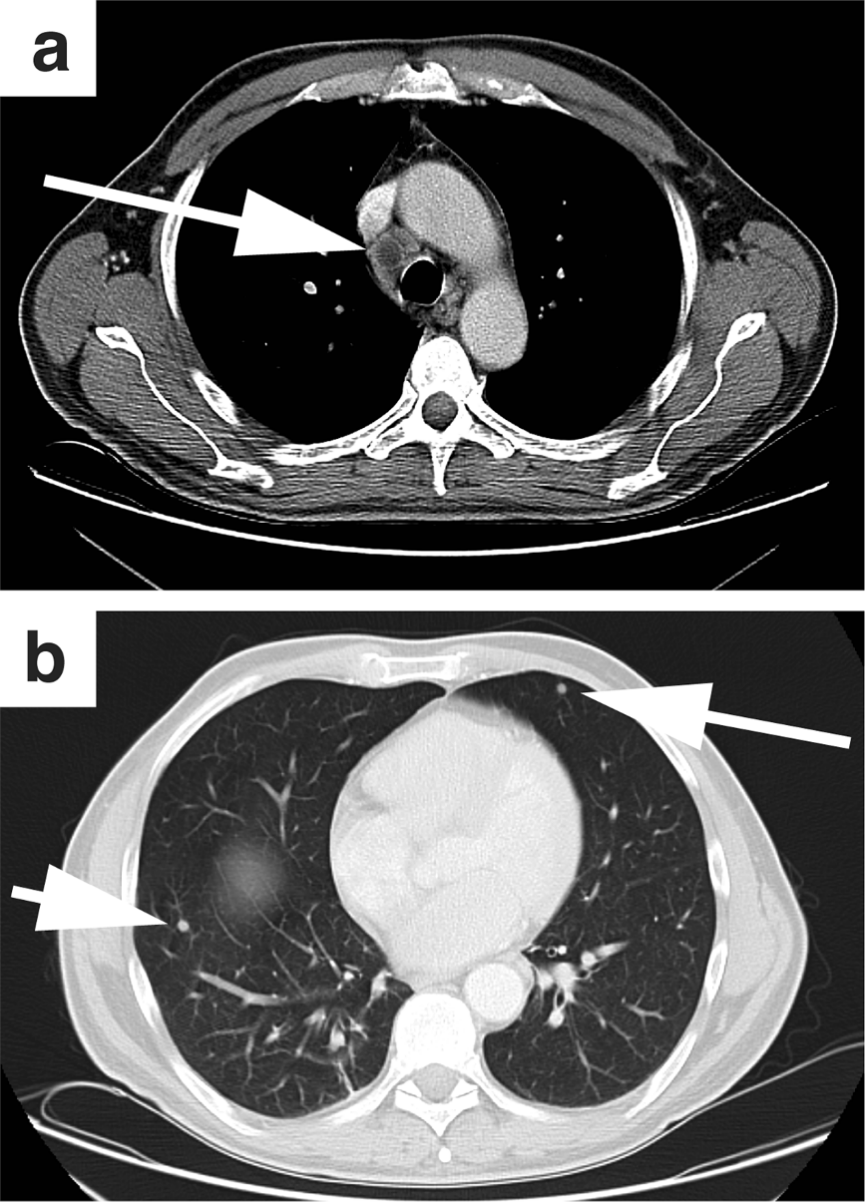
**Radiological diagnosis of disseminated tuberculosis.** CT scan of the thorax on day +43 after allogeneic stem cell transplantation revealed a borderline sized mediastinal lymph node with central necrosis **(a)** as well as multiple pulmonary nodules **(b)**.

On day +26 fever was noted again and antibiotic treatment was re-initiated with piperacillin/tazobactam, which was exchanged for meropenem/vancomycin and then meropenem/linezolid after persistence of fever. Thoracic CT-scan showed multiple small pulmonary nodules and due to the morphology of the lesions pulmonary mycosis and extramedullary leukemia were primarily considered. On day +34 right cervical lymphadenopathy was noted and ultrasonography confirmed three enlarged, inhomogeneous and echopenic lymph nodes with a maximum size of 14×15 mm. Histological analysis of one extirpated lymph node showed complete effacement of its architecture due to multiple epithelioid cell granulomas with only occasional necrosis (Figure 1a,b). In addition, focal polymorphous lymphoid infiltrates (Figure 1c) composed of blastic activated B cells expressing CD20 and CD30 were identified, which were accompanied by plasma cells with polytypic expression of the immunoglobulin light chains. Further immunohistological investigations revealed that the activated B-blasts expressed the EBV encoded latent membrane protein-1 and the nuclear antigen EBNA2, both of which are involved in EBV-induced B cell activation and proliferation (Figure 1d) (Thorley-Lawson 2001). As a sign of a transition of the latent to the lytic EBV infection phase several lymphoid cells expressed the BamHI Z fragment leftward open reading frame 1 (BZLF1)-protein of the virus. Gene rearrangement analysis of the immunoglobulin heavy chains detected the presence of a monoclonal B-cell population. These findings led to the diagnosis of an EBV-associated polymorphic post-transplantation lymphoproliferative disorder combined with granulomatous lymphadenitis. Cultures remained sterile and microscopic examination revealed no acid-fast bacilli. However, PCR analysis of the lymph node revealed presence of Mycobacterium tuberculosis (M. tuberculosis) consensus sequences. Repeated thoracic CT showed mediastinal and hilar lymphadenopathy of up to 20 mm and unchanged pulmonary nodules (Figure 2a,b). Bronchoalveolar lavage was performed and PCR revealed presence of both M. tuberculosis and CMV (191,000 CMV copies/ml). Additionally, sputum samples were found positive for both microscopic and cultural detection of M. tuberculosis. Tuberculostatic therapy consisting of isoniazid, rifampicin, ethambutol and pyrazinamide was initiated. EBV and CMV replication was also detected in the peripheral blood by quantitative PCR (99,300 and 53,800 copies/ml, respectively). Intravenous ganciclovir was initiated and rituximab was administered three times (375 mg/m^2^/dose) with subsequent dropping of copy numbers of EBV and CMV below the detection limits. Two weeks after initiation of TB therapy the patient had completely recovered from all infectious symptoms and sputum samples turned negative for M. tuberculosis. The patient was subsequently discharged from the hospital for outpatient continuation of tuberculostatic therapy. Unfortunately, bone marrow examination on day +77 revealed fulminant relapse of the leukemia without signs of infection. Palliative cytoreduction using hydroxyurea was initiated and the patient was transferred to a hospice, where he died three weeks later. The patient's next of kin consented to the publication of this report.

## Discussion

Fever in patients after allogeneic stem cell transplantation can develop due to various pathogens, including bacteria, viruses, fungi and protozoa. Tuberculosis constitutes a rare infectious complication in patients receiving hematopoietic stem cell transplantation in developed countries. More frequently, active EBV replication occurs in immunocompromised hosts, potentially leading to post-transplant lymphoproliferative disorders (PTLDs), comprising a spectrum from polyclonal B-cell expansion to malignant lymphoma (Swerdlow et al. 2008). Here, we describe a rare case of a German man developing pulmonary and nodular TB as well as EBV-associated polymorphic PTLD and CMV infection after ASCT.

This case features several remarkable issues. TB in ASCT is usually confined to patients from endemic areas (Garces Ambrossi et al. 2005). This case illustrates that TB should be included in an extended differential diagnosis in patients with fever even if from a non-endemic background and if other reasons explaining pyrexia are present. While it is not possible to definitely determine the route of infection in this patient, reactivation of latent TB seems the most likely scenario, because calcified pulmonary granulomas could be noted weeks before clinical onset of symptoms. In addition, the patient reported exposure to TB decades earlier, TB prevalence is low in Germany and reverse isolation measures were observed during neutropenia, making de novo infection less probable. This patient is the first to be diagnosed with TB in a total of 906 patients transplanted at our institution between 2001 and 2012. This low number is likely attributable to the low incidence of TB in Germany. However, fluoroquinolones have proven active in TB and have even been used as TB-prophylaxis by other centers in certain cases (Ip et al. 1998). Therefore, the use of ciprofloxacin as anti-bacterial prophylaxis at our institution could have contributed to the low TB incidence in patient transplanted at our center. However, ciprofloxacin should generally not be considered as first-line tuberculostatic therapy as drug-resistant strains may be encouraged to emerge. Instead, isoniazid is generally considered first choice for empiric prophylaxis of TB in patients undergoing ASCT (Garces Ambrossi et al. 2005).

Several risk factors are associated with TB infection in ASCT patients. Conditioning in our patient included 4 Gy total body irradiation, which has been shown to interfere with alveolar macrophage function besides its immunosuppressive effect on the host in general (Schluger and Rom 1998). In addition, our patient received a graft mismatched at one human leukocyte antigen locus, which has been shown to be associated with higher incidence of TB (Cordonnier et al. 2004). Another frequent risk factor predisposing for TB is GVHD, as it is associated with a delay in T-cell subset recovery. It is likely that therapy for GVHD further increases TB rates, although some controversy remains as to whether the treatment effect on GVHD outweighs its immunosuppressive effect in this regard (Yuen and Woo 2002). However, our patient did not show any signs of GVHD. Increased rates of TB in ASCT patients have also been reported in patients receiving T-cell depleted grafts (Garces Ambrossi et al. 2005). While T-cell depletion was only performed in vivo by administration of ATG, this patient received a relatively low T-cell count, possibly contributing to reactivation of several infections usually prevented by cellular immunity.

Onset of TB related symptoms took place on day +26 after transplantation after recovery of neutrophils. This is in line with previous reports that describe the vast majority of cases to occur after engraftment (Russo et al. 2010), likely due to slow replication times of M. tuberculosis with symptoms developing only after partial immune reconstitution (Yuen and Woo 2002).

There is controversy as to whether and how pre-transplantation screening for latent tuberculosis infection should be performed (Cordonnier et al. 2004). As the tuberculin skin test lacks sensitivity in immunocompromised patients, T-cell-based interferon-gamma release assays have been proposed for ASCT patients (Moon et al. 2012). However, more studies are warranted to establish the role of such assays in the context of ASCT. In addition, screening is only effective in TB endemic areas. Thus, we currently propose not to routinely perform TB screening prior to ASCT in countries not endemic for TB while considering and searching for mycobacterial disease in patients with persistent fever.

In summary, we describe a case of a German male patient developing synchronous TB, EBV-associated lymphoproliferative disorder and CMV pneumonia after ASCT for refractory acute myeloid leukemia. To our knowledge, this is the first case with such a combination of findings. In times of increasing population migration and increased use of T-cell depleted strategies it will be important to bear a high level of suspicion of mycobacterial disease in febrile patients even in countries with traditionally low TB prevalence.
